# Hydration of Workers in Thermal Environments—Practical Recommendation

**DOI:** 10.3390/nu18010064

**Published:** 2025-12-24

**Authors:** Joanna Orysiak, Magdalena Młynarczyk, Joanna Bugajska, Elżbieta Łastowiecka-Moras

**Affiliations:** The Department of Ergonomics, Central Institute for Labour Protection–National Research Institute, Czerniakowska St. 16, 00-701 Warsaw, Poland; m.mlynarczyk@ciop.pl (M.M.); jobug@ciop.pl (J.B.); ellas@ciop.pl (E.Ł.-M.)

**Keywords:** hydration, fluid intake, work, fluid recommendation, hot environment, cold environment

## Abstract

The importance of proper hydration for work performance in hot climates is well known, as opposed to its role in cold climates. Workers’ water requirements may be high in both cold and hot environments, and the effects of dehydration can be a serious problem in either case. The National Institute for Occupational Safety and Health (NIOSH) and the Occupational Safety and Health Administration (OSHA) recommend that workers drink small amounts (150–250 mL at once) of chilled water (especially in hot environments) or warm beverages (especially in cold environments) every 15–20 min (before they become thirsty) to stay well hydrated. However, individual hydration plans are now more preferred, as no single recommendation is suitable for everyone. Workers should stay hydrated before, during, and after work. The article presents the importance of adequate hydration of workers as well as some recommendations for fluid intake in the workplace.

## 1. Introduction

Water is the most important component of the environment consumed by humans. It has long been known that fluid deficiency, leading to disorders of the body’s functioning and death, occurs much faster than the complete lack of access to food [1], and dehydration of more than 15% can lead to death [2,3]. Total body water volume ranges from 45 to 75% of body mass and depends on age, sex, and body composition [2,3,4]. Euhydration is defined as the state of being in water balance. In turn, dehydration (hypohydration) is a deficit of total body water (negative water balance), and hyperhydration (overhydration) is an excess of total body water (positive water balance) [4]. Daily water balance regulation is a dynamic process of body water loss and gain [4].

When working in various environmental conditions, it is very important for the thermoregulation system to function efficiently. Thermoregulation is the ability of an organism to maintain its body temperature within the appropriate bounds (approx. 37 °C ± 1 °C), despite the varying thermal environmental conditions [4]. Depending on physical work (metabolic rate) as well as microclimate parameters, various thermoregulatory processes and mechanisms are initiated in the human body [5], helping to prevent overheating [6,7]. One of the most effective means of heat exchange between the human body and the environment (under favorable conditions) is sweat evaporation [5]. When performing physical work in a thermal environment, the sweat output often exceeds water intake, leading to dehydration of the body [8]. Even 1% dehydration impairs thermoregulation [9]. Despite some recommendations regarding adequate fluid intake in the workplace, deaths due to dehydration continue. On the other hand, overhydration and hyponatremia (a disturbance of the water and electrolyte balance resulting from a reduced level of sodium in the blood serum) are much rarer but may also be life-threatening, especially in the presence of kidney, cardiovascular, or liver disease [2,3,10,11].

Keeping workers well hydrated is one of the most effective interventions to protect their health and productivity [3,8,12]. This is confirmed by The Assessment of Occupational Heat Strain and Mitigation Strategies in Qatar. This project described three strategies for protecting workers against heat stress: adequate (proper) hydration of workers, light clothing, and work organization (work–rest cycle). It was shown that the strategy of proper hydration gave the best results, i.e., the smallest percentage of workers (2%) with an internal body temperature above the norm. Taking into account workers’ subjective assessments, they felt the safest at work during the strategy of adequate hydration (between working hours 6:00–17:00) [13]. Ioannou et al. [12] showed that drinking 750 mL/h of water can reduce the risk of dehydration by 54% and 97% among agriculture and construction workers from Qatar and by 13% among construction workers from Spain. This hydration strategy also resulted in a significantly reduced core temperature, but there was no effect on labor effort [12].

Providing portable water is the responsibility of employers [14,15,16]. However, despite this, many workers do not consume adequate amounts of fluids at work [17,18]. Workplace interventions to improve workers hydration should include engineering and administrative controls in close cooperation with employers [18]. Strategies used by employers that can reduce dehydration among workers include, for example, education, adequate access to safe drinking water (e.g., through the use of water bottles), drinking fluids during rest breaks, health monitoring systems, access to clean toilets, hydration backpacks, and rest in the shade or use of cooling technologies in hot environments [19,20,21].

Despite the implementation of various strategies, dehydration remains a serious problem for workers exposed to both hot and cold environments [22,23,24]. Orysiak et al. [24] showed a higher percentage of dehydrated foresters in winter than in summer. Furthermore, many workers are inadequately hydrated before and after work, regardless of the season [18,24,25,26].

Proper hydration is essential for health, safety, and work performance [8], yet it is often neglected and under-researched [20].

Studies have shown poor hydration status in workers from various ethnic, cultural, and economic backgrounds working in various industries [27]. That is why it is so important to ensure proper hydration of workers as well as their education in this area. The purpose of this narrative review was to provide key information regarding dehydration, including factors influencing water needs, fluid consumption strategies, as well as fluid types and their impact on hydration status. This information can be used by employers to develop appropriate workplace strategies to optimize workers’ hydration.

### Review Methods

The review method used in this study involved searching online databases of Polish- and English-language scientific journals in PubMed and Google Scholar using the following keywords: “hydration”; “dehydration”; “hydration status”; “sweating rates”; “workers”; “population”; “athletes”; “soldiers”; “monitoring”; “hydration status markers”; “hydration status indices”; “hot microclimate”; “cold microclimate”; “heat stress”; “cold stress”; “water”; “beverages”; “total water intake”; “fluid intake”; “water intake”; “drinking strategies”; “water requirements”; “hydration recommendation”; “OSHA”; “NIOSH”; and “EFSA”.

The study took into account original and review articles as well as books and websites of various organizations written in Polish and English published until 2025 (inclusive).

## 2. Factors Affecting Water Requirements

The European Food Safety Authority (EFSA) recommends that the adequate total water intake (AI) for adults per day is at least 2 L for women and 2.5 L/day for men [28], including water from drinks, products, and dishes. However, approximately 80% of the consumed water should be supplied in the form of liquids [28]. Studies showed that 60% of men and 40% of women do not consume the amount of water from fluids consistent with the EFSA recommendations, which may lead to dehydration [29]. Moreover, deeper analysis of total water intake reveals that 16% of men and 4% of women are below the EFSA recommendation on every testing day [30]. Workers also often not only become dehydrated at work but start their working day in a hypohydrated state [8]. However, it should be noted that reaching the recommended level of water does not mean that a person is consuming enough fluids to meet their needs [31]. The requirements for fluids are most often underestimated, and most people working in difficult conditions and/or who are physically active take too little fluids [1]. An inadequate hydration status may have a negative health and cognitive impact on daily life at work and at home in workers, especially if they are not properly hydrated for several days per week [30].

Water requirements depend on many different factors.

Below, the factors are divided into internal factors and external factors.

### 2.1. Internal Factors

**Figure 1:**
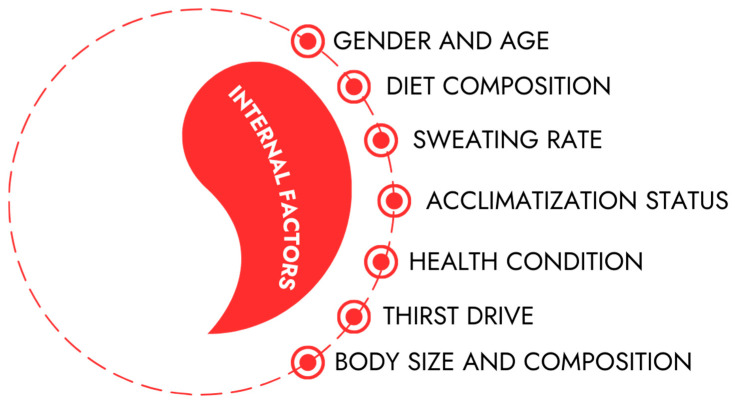
Selected internal factors influencing water requirements.

Selected internal factors that depend on human nature and lifestyle are described below (Figure 1):

- GENDER—Due to differences in body mass and metabolic rate between men and women, men have a higher daily fluid requirement (higher sweating rates). However, the mean sweating rate (expressed relative to body surface area) is similar between women and men in temperate and hot, dry conditions, but is lower in women than men in hot, wet conditions. Less sweat is wasted as a result of the greater suppression of sweat (due to wetted skin). Under these conditions, women lose less fluid because of a lower sweating rate. Men produce more sweat in humid conditions and become more dehydrated (wasting water because of difficult evaporation and insignificant effect on cooling) [2,32,33,34,35,36,37].
- AGE—The influence of age is noticeable both on the thermoregulatory capacity and on the regulation of fluids in the body. Heat tolerance deteriorates with age. Older people begin to sweat later (and less effectively) than younger people, which causes older people to increase their core body temperature or weighted average skin temperature to a greater extent. It should also be noted that the feeling of thirst decreases with age. Older people also have lower total body water content and reduced kidney function [4,38,39,40,41,42,43,44].
- DIET COMPOSITION—The requirement for water also varies depending on the diet composition. More water should be provided as the energy value of the diet increases (because larger amounts of nutrients must be metabolized), and a diet high in protein increases the removal of water-soluble nitrogen compounds (leading to increased diuresis). Excessive fiber consumption promotes greater water loss through feces. In turn, insufficient supply of carbohydrates (CHO) also increases the water requirement due to the need to remove ketones in the urine [3,45,46].
- SWEATING RATE—Sweating rates can depend on work intensity and duration (increasing in proportion to both) as well as heat acclimatization (which increases sweating), fitness levels (higher fitness level increases sweating), clothing (higher clothing insulation increases sweating), and ambient temperatures (higher temperature increases sweating). On the other hand, wet skin (from high humidity) can reduce sweating. The amount of sweat lost varies individually, but usually women produce less sweat and also lose fewer electrolytes than men [1,3,4,31,36].
- ACCLIMATIZATION STATUS—Acclimatization to a hot environment by exercise of low to medium intensity in high temperatures for 1 h a day (or longer) over 9–14 days reduces the risk of heat stress. Heat acclimatization increases sweating rates and therefore requires greater fluid intake; however, heat-acclimatized people drink more often, and they can more accurately match fluid intake to the amount of sweat lost. Changes in the intensity of sweating occur between the 3rd and 10th day of exposure [2,47,48].
- BODY SIZE AND COMPOSITION—The amount of sweat produced is almost proportional to the two-thirds power of body weight, so a 91 kg man will sweat about 30% more than his 59 kg companion performing the same intense exercise. Water constitutes approximately 40–70% of human body weight, and its total content depends on gender, age, and body composition (lean body mass contains ~70–80% water, whereas adipose tissue contains ~10%). Therefore, people with low body fat have a higher total body water content than people with higher body fat, even with the same body weight [2,3,8,35,36,49].
- THIRST DRIVE—The physiological onset of thirst occurs when a person is slightly dehydrated, starting at a weight loss of 1–2%. Moreover, thirst can be suppressed by cold exposure as well as intense physical effort at work [22,36,50,51,52,53,54,55].
- HEALTH CONDITION—Fever, vomiting, or diarrhea increase fluid losses, so water requirements increase during this time. Moreover, certain drugs, for example, antihypertensives, hypolipemics, hypoglycemics, or drugs for acid- or nervous-related disorders, may affect hydration status. In addition, diabetes mellitus, obesity, or renal disease can also induce water balance alterations [28,45,56,57,58,59].

### 2.2. External Factors

**Figure 2:**
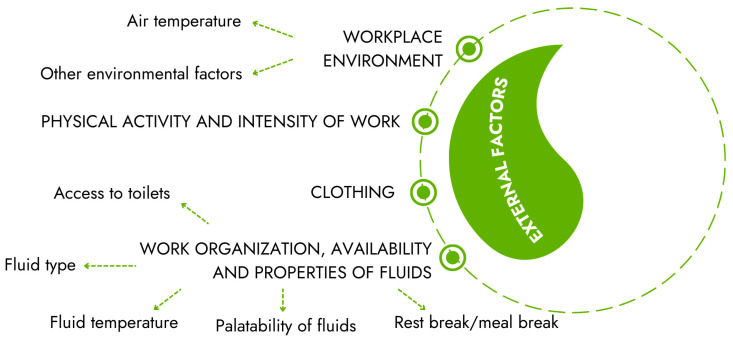
Selected external factors influencing water requirements.

Selected external factors that depend on the environment and are directly related to the workplace are described below (Figure 2):

- WORKPLACE ENVIRONMENT:
  1. Air temperature—When workers work in unfavorable temperature conditions, fluid intake increases depending on environmental conditions. The water requirement increases not only in warm environments at elevated temperatures but also at low temperatures or high altitudes. Water loss from the body—mainly through sweat—significantly increases in warm conditions (sweating begins above 28 °C). In such environments, the rate of sweating (water loss) can reach approximately 2–3 L/h, which is the main cause of dehydration in hot conditions. Water requirements may increase also from 4 to 6 L/day and from 8 to 10 L/day when workers or soldiers perform heavy physical work or for many hours moderate physical work in a moderate environment and in an extremely hot environment, respectively. Some studies have shown that, for soldiers, miners, or construction workers, water losses and therefore also fluid requirements can reach 10–12 L/day in a hot environment. Dehydration in cold environments may reach 3–8% of body weight (levels are similar to those reported for workers in hot climates). The most important factors causing dehydration during work in cold environments are cold-induced diuresis, respiratory water losses, cold-protective clothing (sweat loss due to overdressing), the metabolic cost of movement, impaired thirst sensation, reduced desire to drink, limited access to fluids, and reduced fluid intake to minimize urination [2,3,8,22,31,36,60,61].
  2. Other environmental factors (the influence of air humidity, air movement, sunlight, or radiation temperature)—The level of air relative humidity may affect the demand for water. At low relative humidity and elevated air temperature, the need for water increases due to increased sweating and the rapid evaporation of sweat from the surface of the skin; air movement also supports heat dissipation and the effectiveness of sweat evaporation. Moreover, high humidity in warmer environments may increase water requirements, although wet skin (from high humidity) can reduce sweating. It should also be noted that cold, dry conditions may increase water loss from the respiratory tract when breathing cold and dry air. Extreme thermal working conditions, which may affect the occurrence of dehydration in workers, are also sometimes observed in atypical places. For example, some buildings (such as hospitals) are of a design type that makes them susceptible to overheating with often hot and humid environments. Moreover, if relative humidity falls due to the use of air conditioning without humidification, an increase in respiratory water loss can occur. Thus, maintaining optimal working environmental temperatures and humidity may help reduce sweating and consequently fluid loss [5,22,23,36,61,62].
- CLOTHING—Clothing is another factor that can have a significant impact on daily water requirements. Typically, garments provide insulation and inhibit vapor evaporation, increasing the heat load, thereby raising the sweating rate compared to wearing shorts and t-shirts. However, in some conditions, clothing can help reduce water requirements. For example, in a hot, dry environment (desert, full sun), light, “breathable” solar-reflecting material can reduce perspiration by up to 20%. On the other hand, in a cold environment, water loss can be the effect of heavy and cumbersome clothing, which causes significant heat storage and sweating. Soldiers dressed in cold-protective clothing (with high thermal insulation ~ 4.0 clo) produced little sweat while resting in the cold, but lost nearly 2.0 L of sweat per hour during moderate or heavy exercise. However, when thermal insulation was about 1.9 clo, a five-fold reduction in the rate of sweating was observed (only approximately 0.4 L/h). Workers in cold climates should dress in layers. Additionally, using face masks can also impede proper fluid intake [2,3,8,22,23,61].
- PHYSICAL ACTIVITY AND INTENSITY OF WORK—The intensity of exercise and its duration reflect the rate of sweating and thus the total loss of fluid. The water requirement increases with greater physical activity (increased losses of water from sweat and through the lungs are observed with longer and more intense efforts). Depending on exercise intensity, duration, and type, fluid requirements vary. Occupational work is characterized by different types of activity (aerobic, anaerobic, and/or strength tasks) interspersed with rest breaks, so large sweat losses in workers can occur, especially when combined with protective clothing and/or extreme thermal work conditions. Moderate-intensity military tasks are estimated to produce a sweating rate of approximately 0.3 L/h, while high-intensity work increases the rate of sweating to approximately 0.7 L/h. However, if moderate-intensity work (with a sweating rate of 0.3 L/h) is extended to 8 h, the daily fluid requirement increases by an additional 2.4 L/day. If people do not match their fluid intake with their sweat loss, it can lead to over-drinking or under-drinking during different types of physical activity [1,2,3,22,23,31,36,47,63,64,65,66].
- WORK ORGANIZATION:
  1. Access to toilets—Some people, especially women, will intentionally not drink fluids when toilets are not easily accessible or unhygienic [8,26,54,67,68].
  2. Water availability—It is worth emphasizing that if water is not available to workers, they will not be able to maintain proper hydration in the workplace, and this may have a negative impact on their health and productivity at work [19,21].
  3. Rest break/meal break—Workers will refrain from drinking water if they have to walk long distances to get it. Workers may become voluntarily dehydrated while working, especially in cold or hot climates, but they may replenish water with meals. Meals are a valuable opportunity to maintain hydration (as they stimulate thirst and result in the intake of extra fluids) and provide nutrients (including electrolytes lost through sweat) necessary to achieve full hydration. It was shown that 30% of water is obtained from food in total in-shift fluid intake of workers [2,69].
  4. Fluid type—Water is the most commonly consumed beverage throughout Europe. However, drinking patterns and quantities vary and are influenced by age, gender, diet, physical activity level, time of day, environment, and climatic conditions, as well as the availability of palatable tap water or other drinks, regional differences in drinking pattern culture, and tradition. As beverage preferences may vary among employees, workers should have a certain degree of “autonomy” regarding the type of fluids they consume [30,54,70,71,72,73,74,75,76].
  5. The palatability of fluids—Odor, taste, temperature, color, and fluid turbidity influence fluid palatability. Voluntary consumption of fluids will be reduced if the water has an unpleasant taste or odor. Allowing workers a certain degree of “autonomy” regarding the palatability of the fluids they consume could maximize enjoyment and help increase fluid intake [2,70,75,77].
  6. Fluids temperature—Some authors described that drinking too cold drinks can cause cramps in the digestive system and, due to the constriction of blood vessels and reduced blood flow, also slower fluid absorption. Therefore, they recommend beverages close to body temperature. However, such an approach does not seem to be justified, especially in a hot and humid microclimate, because the regulation of gastric emptying is multifactorial, with the basic factors being gastric volume and energy density of the drink (high energy density and small stomach volume slow emptying rates). Drink temperature has little effect on gastric emptying. The National Institute for Occupational Safety and Health (NIOSH) and the Occupational Safety and Health Administration (OSHA) recommend that in hot environments, workers should drink chilled fluids (10–15 °C, not too cold), whereas in cold environments, it is better to choose warm drinks. A chilled drink could act as a “cooler” because it requires an energy input to raise the temperature of the ingested fluid to body temperature, and this suppresses the increase in internal temperature. In turn, in a cold environment, when body temperature is lowered, drinking warm beverages may help increase core body temperature. On the other hand, it was suggested that drinking hot beverages may “cool” the body, if sweat can evaporate from the body’s surface. This happens, for example, in a dry and hot environment. In the case of high temperature and high humidity, sweat evaporation is difficult, and thus hot drinks do not provide a cooling effect [1,23,31,68,70,75,78,79,80,81,82,83,84,85,86].

Many factors influence water requirements, some of which can be managed by both workers and employers, while others are beyond their control. When determining water requirements, all factors should be considered to tailor fluid intake to individual needs.

## 3. Hydration Status Among Workers

Workers often carry out heavy physical work for many hours, with relatively short rest breaks and sometimes with limited fluid intake [31]. Dehydration is very common among workers both at the beginning and/or at the end of work in various industries, such as in fire brigade, forestry, industry, mining, agriculture, and construction [24,26,69,87,88,89,90,91,92,93,94,95,96] (Table 1). As mentioned earlier, workers working in thermally variable environments have been observed to have poor hydration status throughout the year [24]. Likewise, among those who predominantly performed sedentary work in cool or moderate environments (e.g., teachers, office workers, security guards), a significant proportion of workers were dehydrated, and many remained dehydrated at the end of their shift [67]. Moreover, a significant proportion of doctors and nurses were dehydrated at the start and end of medical and surgical shifts, and many of them were oliguric [62].

## 4. Consequences of Dehydration

Insufficient fluid intake can lead to dehydration, which can seriously affect human health. The symptoms of dehydration are shown in Table 2 [101,102,103].

During physical activity at work, metabolic heat is generated through muscle contraction, which can lead to hypovolemia (decreased plasma/blood volume) and thus to cardiovascular stress, increased glycogen use, altered metabolic and central nervous system function, and a greater increase in body temperature [60]. The additional thermal strain associated with dehydration may contribute to the increased risk of heat illness [60]. The response to dehydration is complex and individual [60]. Even slight dehydration (1–2%) among workers can negatively affect their ability to work and their performance [2,8,90]. The greater the dehydration, the more serious its effects, which may be dangerous to the health and lives of the workers [9] (Table 3).

Dehydration can impair not only job performance but also workers or other people’s health and safety [8,36]. Coombes et al. [106] described that thirst, hunger, and tiredness were the main causes of prescribing errors by interns [106]. This highlights the importance of proper hydration given that one of the main tasks of interns is to prescribe and administer drugs [62]. Therefore, maintaining adequate hydration in the workplace is especially important, as dehydration can negatively affect productivity, safety, costs, and morale at work [8].

Importantly, not only a water deficit but also its excess can be harmful, especially when large amounts of fluids are consumed at once, significantly exceeding the kidneys’ maximum water removal capacity of 0.7–1.0 L/h [28,46]. Excessive fluid intake is a major etiological factor in exercise-associated hyponatremia, so using the innate thirst mechanism to guide fluid intake is a strategy that can help prevent overdrinking and development of hyponatremia while providing sufficient fluid to avoid excessive dehydration [1,107].

## 5. What We Lose with Sweat

It is very important to remember that during the day, and especially after exercise/work, not only fluids but also electrolytes—which are lost through sweat—should be replenished. When people rehydrate with plain water, drink more water than they have lost in sweat, or do not consume enough electrolytes, sodium deficiency (hyponatremia) may develop [1]. It was found that among South African forestry workers harvesting trees, overhydration was observed in 23% of them in autumn and 13% in winter before work, as well as in 4% in autumn and 2% in winter after work [26]. Workers consumed large amounts of hypotonic fluid (water) in the forest. In addition, workers were instructed to drink as much water as possible to prevent dehydration, as it was assumed that excess fluid would be removed in the urine. Cooking with salt (NaCl) and adding it to prepared food was also actively discouraged to prevent the development of hypertension. Excessive hypotonic fluid intake, combined with restricted salt consumption, may contribute to the risk of potentially fatal dilutional hyponatremia in some workers [26]. After intense physical work, especially in hot weather, significant sweat loss is observed. In such situations, sodium restriction in the diet should not be avoided, as sodium losses caused by heavy sweating need to be replenished [108].

Through sweat, people principally lose sodium and chlorine and, to a lesser extent, potassium, calcium, magnesium, and some vitamins [1,31,36,45]. The total amount of sodium lost depends on the sweating rate and duration as well as its concentration in sweat. The composition of sweat varies individually and, in addition to genetic predisposition, depends on factors such as diet, dehydration, and the degree of acclimatization [35,36,109,110,111,112]. Notably, high sweating rates (≥2 L/h) or long periods of intensive exercise may increase sodium loss even in people with low or average sweat sodium concentration [36]. Heat acclimatization decreases sodium chloride concentration for a given sweating rate due to improved sodium chloride reabsorption [36,111,112]. Bates and Miller observed that during a 10 h shift under moderately hot conditions (35 °C, 50% RH, 40% V_O2_max), the mean sodium losses were 4.8 g for acclimatized workers and 6 g for non-acclimatized workers, corresponding to 10–15 g salt [113]. The excretions of water-soluble vitamins and minerals in sweat during an eight-hour work shift among heat-exposed steelworkers increased with rising temperature in the work environment [114]. It is worth emphasizing that both fluid and electrolyte losses due to prolonged sweating should be compensated for to prevent imbalances in water and electrolyte homeostasis [45,113]. The amount of sweat lost does not correspond directly to the number of electrolytes lost [115]. The general public tends to consume more salt in their daily diets, so there are usually no salt deficiencies [116]. However, in the case of intense sweating and large losses of salt, it is advisable to “supplement” salt in the form of additional salting of food, salty snacks, or drinking beverages with electrolytes. Salt tablets are not recommended [45,109,117,118]. Excess electrolytes or vitamins must be eliminated in the urine, which also causes water loss and reduces hydration efficiency. Consuming too much salt can also cause nausea or vomiting, which can further exacerbate dehydration [1,118].

## 6. Fluid Consumption Strategies

In the case of competitive athletes, the literature describes various strategies for fluid consumption before, during, and after exercise, which are important both for health and for optimizing exercise capacity (Table 4) [119,120]. However, for the general population, no such precise guidelines exist [3].

It is generally believed that, as a result of ad libitum access to food and drink, water losses (approximately 1% of body weight) are usually compensated overnight (within 24 h) [8,28]. Daily fluid intake, driven by a combination of thirst and consumption of drinks with meals, keeps people hydrated and at a normal level of their total body water, even though temporary water deficiencies may occur a few hours after reduced water intake or increased sweat losses (physical activity, environmental exposure) [3,31,121]. This is confirmed by several studies in which, despite dehydration experienced by workers during their daily work, proper hydration status was observed the following morning. However, this outcome is only possible if progressive hypohydration is avoided during the working week [31]. When significant body water losses occur, it can sometimes take several hours of adequate fluid and electrolyte intake to restore the body’s water balance. For example, if workers experience dehydration > 4% of their total body weight, fully rehydration may require more than 24 h [3,8].

In scientific studies among athletes, no differences were found in the effect of various drinking strategies during training on physical performance [3,119,122]. However, these studies were rarely conducted in cold or hot environments. In environments where exposure to thermal stress is very high (with a significant sweat rate), some authors suggested that regular drinking seems to be more appropriate for workers than voluntary and/or thirst-driven drinking (at least until workers develop appropriate drinking practices) [27].

Although there are no precise guidelines regarding the amount and timing of fluid intake at work, some institutions have issued recommendations for fluid intake in the workplace. According to OSHA [84,85,118] and NIOSH [82,83,117], workers should frequently drink small amounts of water or warm beverages (especially in cold environments) before they become thirsty to stay well hydrated. Workers should start drinking at work as early as possible and drink frequently, in small portions (150–250 mL at one time; fluid intake should not exceed 6 cups/hour, or approximately 1.5 L/h) [3,82,83,84,85,117,118]. It is not recommended to drink more than 300–400 mL at one time [1] or to drink excessively in order to gain weight [120]. However, in some work conditions (e.g., wearing personal protective equipment), frequent drinking is not practical. In such situations, workers should drink enough fluids before work and during breaks; for example, it is recommended to drink 500 mL of water/fluids per hour before work and 500 mL of water/fluids during rest periods [115,123]. Moreover, it is much more beneficial to regularly, frequently drink small amounts of fluids (improving fluid tolerance and utilization) than to wait an hour or more and drink all fluids at once (e.g., a liter of chilled water), as this can lead to nausea, vomiting, and headache, especially under high thermal stress [3,22,115].

The above recommendations are very general, and it should be remembered that human water needs can be highly variable [115]. Therefore, no single recommendation will be suitable for everyone, and each worker should monitor his/her own fluid losses and create an individual hydration plan [115,124,125,126,127]. Recommendations for optimizing fluid intake should be based on individual sweating rates or, in some cases, on individual thirst [124]. More personalized fluid intake may also be specified according to body mass [115]. Clinical calculations usually estimate water requirements using equations such as mL/kg body mass, by 10 kg weight categories, percentage of body mass, surface area (mL/m^2^), and ml/kcal expenditure [128,129].

Water requirement [128,129]:

- Determined in mL/kg (Equation (1)):

Water requirement = (30–45 mL)*/1 kg body mass(1)

* Current estimates for adults depend on age, sex, and body mass, and vary between 30 and 45 mL/kg/day.

- Determined in mL/kcal expenditure (Equation (2)):

Water requirement = 1 mL/1 kcal(2)

Workers often do not have established individual hydration plans and, therefore, may consume either excessive or insufficient amounts of fluids. In a study of foresters, some of them had a > 1% increase in body mass after work, indicating that they may have consumed too much fluids. Lower urine specific gravity and higher total body water after work compared to before work support this assumption [24]. Moreover, discrepancies in the amount of fluid intake between workers were observed [17,26,67,130]. Orysiak et al. 2023 [17] described that the amount of fluids consumed by foresters ranged from 250 mL to 2500 mL in the summer and from 350 mL to 1800 mL in the winter. These studies confirm that workers do not have individual hydration plans and do not consume fluids according to their needs [17,130].

Fluid intake during work was appropriate for most workers to maintain, but not improve, hydration status [27,31]. This is because, in some thermal environments, the rate of sweat loss can be as high as 1 L/h or even exceed it, which is close to the maximum rate of fluid absorption from the gut [27,87,89]. That is why it is so important to start work well hydrated and to maintain hydration with a proper drinking schedule [27]. However, it should be emphasized that workers can stay well hydrated when working in the heat. Miller and Bates showed that high awareness of adequate hydration among workers allows them to stay hydrated at work [89]. Manual workers (construction), despite experiencing the highest sweat losses (>1 L/h) among all studied groups of workers, were the best hydrated and showed minimal weight loss during work. These workers consumed, on average, almost 9 L of fluid during work. The use of physiological adaptation to the hot environment, by combining adequate hydration with a work pace adapted to thermal conditions (self-pace policy), allows workers to safely continue working even in the most extreme thermal conditions [27,131]. On the other hand, drinking fluids according to OSHA recommendations (3–4 cups of approximately 240 mL/h) is insufficient to maintain hydration in farmworkers [132].

However, workers are usually not able to fully replace fluid losses, so it is very important to drink properly afterwards, especially when dehydration is significant. If workers have sufficient time before starting their next job, consuming normal meals and drinks will restore hydration [35]. Nevertheless, some authors have suggested that, due to the persistent sweating process and obligatory urine loss, effective rehydration requires more fluid intake (e.g., 125–150%) than the final fluid deficit (e.g., 1.25–1.5 L of fluid for each 1 kg of body weight lost) [60]. Similarly, when aggressive and rapid replacement of fluid losses is needed, the consumption of fluids and electrolytes to compensate for 100–150% of weight loss will allow for adequate hydration [53]. However, care should be taken to avoid overhydration [1].

**Figure 3:**
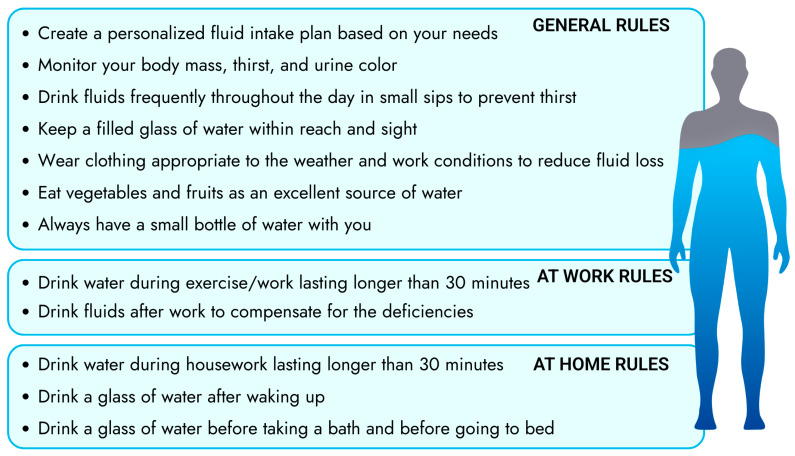
Proposed strategies for increasing fluid intake [8,47,78,133].

As emphasized earlier, the consumption of fluids not only during work, but also throughout the day, is very important. After work, fluids should be consumed in combination with sodium-containing meals (especially in the case of heavy salt sweaters) throughout the day [115]. Below is the authors’ proposition for a daily fluid intake (Figure 3).

It is also important to drink fluids before work (particularly physical work) to keep adequate blood flow through the gastrointestinal tract (keeping the stomach and intestines partially full) and to prevent drastic redistribution of blood to the working muscles that occurs when the gastrointestinal tract is empty [1]. In order to achieve good hydration, it is preferable to maintain a high gastric volume, as the rate at which fluid leaves the stomach decreases as stomach volume decreases. Several factors can negatively affect gastric emptying and fluid absorption. Hypohydration ≥ 3%, as well as high exercise intensity (>∼70% to 75%), slows gastric emptying and intestinal water absorption. A high rate of gastric emptying can be maintained by keeping a high gastric volume through frequent consumption of small amounts of fluid during work [134,135].

How can workers check how much water they need during work? The most important thing is to ask themselves how much sweat they lose during work, because sweat production has the greatest impact on water balance during work [1,31,36]. The simplest method for assessing fluid loss is to weigh oneself before and after work (without sweat-soaked clothing). These calculations should be adjusted for food and drink consumption and urine/fecal loss [1,35,136]. Monitoring hydration status before work should be based on urine color, thirst, and weight changes [8]. However, during work, workers should monitor urine color and toilet use frequency—urine should be light yellow, and they should use the toilet at least once during work [17,19,25].

## 7. Types of Fluids

**Figure 4:**
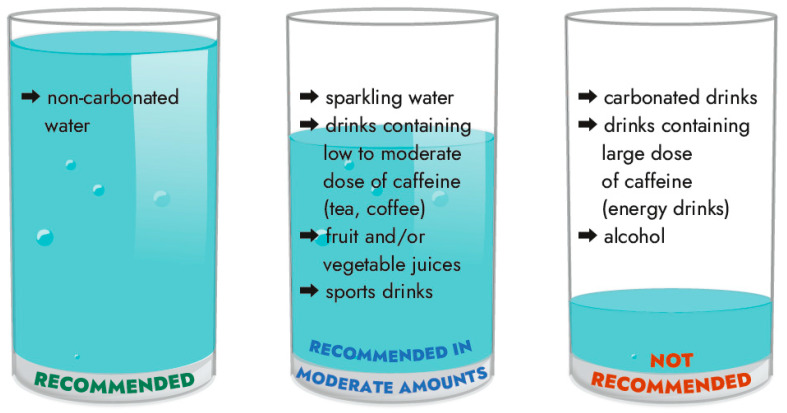
Recommendations for the consumption of various types of fluids at work [17,31,45,82,83,84,85,89,117,118,133,138].

Drinking patterns and fluid intake among workers have not been examined extensively. Available literature suggests that water is the most consumed beverage in the workplace [17,54,95], but soft drinks and/or coffee are also often consumed [17,54,95]. The type of drink workers choose depends on the loss of water and electrolytes during their work. Fluid selection at work should take into account caloric requirements, nutrient content (dissolved) such as carbohydrates, electrolyte content, palatability, and beverage availability [137]. According to the recommendations, workers at work should drink (Figure 4):

- Non-carbonated (still, plain) water—Workers should consume the largest amount of non-carbonated water (spring or mineral). Spring water is universal, while mineral waters require understanding of individual health needs. For example, people with kidney stones should avoid water with a high calcium content, whereas people with osteoporosis should especially choose this type of water. People suffering from hypertension should select low-sodium water, while workers exposed to high temperatures may benefit more from water with a high sodium content. However, one should not drink large amounts of water (without sodium at the same time) because it is hypotonic and is excreted faster by the kidneys, accelerating dehydration of the body. Moreover, excessive water consumption may disturb the water—electrolyte balance [31,45,82,83,84,85,89,117,118,133,138,139].
- Fruit and vegetable juices and/or sports drinks—Workers should drink these in limited amounts. They contain carbohydrates and other nutrients that may be beneficial in certain situations. However, consuming these drinks in the amounts required to compensate for sweat loss may cause gastrointestinal discomfort due to the slowing of gastric emptying, which is proportional to the energy content of the drink. In addition, sports drinks (isotonic drinks) and fruit juices can increase total energy consumption during the day and therefore should not be consumed in large amounts [31,79,89,117,118,137].
- Carbonated drinks—Carbonated (especially sugary) drinks should be avoided because the carbon dioxide (CO_2_) they contain stretches the stomach walls and reflexively inhibits thirst, which may lead to workers drinking less fluid than needed. Moreover, these drinks contain added sugars, which may increase calorie intake. However, Maughan et al. [140] reported that sparkling water is as hydrating as regular water. On the other hand, apart from carbonation’s effects on satiety, sparkling water and other carbonated drinks may affect bloating and may worsen this condition. In general, some workers may find the fizz from CO_2_ appealing, which can increase their daily water intake. The Centers for Disease Control and Prevention (CDC; USA) promotes water, even sparkling water, over sugary drinks for those who do not like to drink plain water. However, waters containing CO_2_ are not recommended for people suffering from hyperacidity, gastric and duodenal ulcers, throat and vocal cord diseases, as well as people with respiratory or circulatory failure. Moreover, if workers do not consume enough calories or if they are not hungry at mealtimes, they should not choose carbonated water, as it may further reduce their food and fluid intake [31,45,140,141,142,143,144,145,146,147,148,149].
- Drinks containing caffeine—Some authors recommend avoiding drinks containing caffeine because they can increase diuresis and the loss of larger amounts of water and disrupt the body’s water and electrolyte balance. According to the EFSA [150], daily caffeine intake from all sources up to 400 mg (approximately 5.7 mg/kg body mass) seems safe for adults in the general population (except for pregnant women). Large doses (≥500 mg) of caffeine may elicit a diuretic effect. On the other hand, the development of tolerance to the diuretic effect may appear with regular coffee drinking. However, four days is sufficient time for the loss of tolerance when caffeine is discontinued. The common opinion is that higher doses of caffeine—but not low to moderate doses—may cause an acute increase in urine volume in caffeine-naive individuals (those who do not habitually consume caffeine or those who have abstained from caffeine consumption for ≥ 4 days) [151,152,153]. Because of that, some recommendations state that coffee or tea can effectively contribute to total daily fluid intake [64,117,118,138,154].

To improve hydration, workers should drink fluids at a faster rate than their loss, but this is difficult when the average sweating rate is above 1 L/h. The ability to consume more fluids to improve/maintain hydration may be limited by the maximum fluid absorption rate (~1.5 L/h). Therefore, some authors have suggested that in this case, rehydration fluid that is rapidly absorbed and has fluid retention capacity may play a useful role. However, such a fluid should be “designed” for use by industrial workers rather than athletes and should be capable of being consumed in large amounts over long periods of time [89]. Products intended for athletes are often high in carbohydrate content, as maintaining blood glucose levels during strenuous physical activity is extremely important. However, for ‘regular’ workers, the main goal should be to consume large amounts of fluid during an 8–12 h shift at work, which can lead to over-consumption of calories if a high-carbohydrate product is used. Moreover, drinks with lower carbohydrate content are considered tastier with longer consumption [27]. Sports drinks with excessive carbohydrate content can cause gastrointestinal upset during physical exertion. Sports drinks with sweeteners can cause even greater gastrointestinal discomfort than isotonic drinks with free sugars [155]. On the other hand, the maintenance of physical and cognitive function in workers is also important. Adding carbohydrates helps maintain blood glucose concentration and supplies fuel to the muscles and brain during prolonged work. Cuddy et al. [156] determined the impact of supplemental liquid or solid carbohydrate feeding on self-selected activity during wildland fire suppression. They concluded that carbohydrate feeding significantly increased average activity levels throughout the day as well as per minute activity 2 h before lunch and in the last 4 h of the workday compared with a placebo. Therefore, work output—especially during the latter hours of the workday—is increased by consuming liquid and/or solid supplemental CHO [156]. However, a fluid-replacement beverage should contain an amount of carbohydrate that depends on the workers’ energy demands [36]. On the other hand, adding additional electrolytes to the fluid mixture may reduce the amount of fluids that need to be consumed, which may be beneficial in situations where water availability is limited [157]. However, this issue requires further research.

The most common form of fluid replacement is drinking plain water. However, sometimes to improve palatability, stimulate thirst, accelerate intestinal fluid absorption, and promote fluid retention, the beverage should include carbohydrates, electrolytes, and/or other ingredients. The beverage composition may depend on the source of fluid loss (sweat, urine, respiration, or diarrhea/vomiting), the population, and environmental conditions [36]. It was reported that 98.5% of American farmers consumed water at work, but there was also a high consumption of sodas (48.2%), sports drinks (46.2%), and juices (24.9%) [158]. Therefore, it is important to be aware that the consumption of any beverage contributes to workers’ total fluid intake, and advice on avoiding certain drinks (e.g., those containing caffeine) may result in lower overall fluid intake when these drinks are part of normal dietary practice [137,140]. However, it is essential to understand the health effects of individual beverages, as some are not recommended for daily consumption [154,159,160].

## 8. Workplace Hydration—Recommendation

There are currently no specific recommendations for fluid intake in the workplace that take into account environmental influences, protective clothing, and work intensity. Hydration and work pacing guidelines that include work intensity, environment, work–rest cycles, and fluid intake were developed by the U.S. Army [161]. It is important that such recommendations for employers and workers are also developed in the future, but if they are too general, they may recommend too much or too little fluid consumption depending on the environment, individual predispositions, and work intensity [8]. Establishing and maintaining a culture of hydration awareness among workers may require changing habits related to time, type, and amount of fluid ingestion, with the aim of starting work in a well-hydrated state and maintaining this by replacing fluid to keep pace with sweat losses [27].

Employers are obligated to provide workers with water [14,15,16].

For example, in Polish law, the obligation to provide workers with potable water or beverages is defined in the Regulation of the Minister of Labor and Social Policy on general occupational health and safety regulations (JL 1997, No. 129, item 844; as amended) [16]. According to the regulation, “The employer is obliged to provide all workers with potable water or other beverages, and to provide workers permanently or periodically employed in particularly difficult conditions with other beverages in addition to water. The quantity, type, and temperature of these beverages should be adapted to the working conditions and the physiological needs of the employees. Drinking water sources should be located no further than 75 m from workstations.” Detailed rules for providing beverages to workers employed in particularly difficult conditions are specified in the Regulation of the Council of Ministers on preventive meals and beverages (JL 1996, No. 60, item 279, as amended) [162]. According to the regulation, employers are obligated to provide beverages to workers in quantities sufficient to meet their needs, either cold or hot, depending on the working conditions. Employers must provide beverages to workers working:

(1) In hot microclimates (WBGT above 25 °C),
(2) In cold microclimates (WCI above 1000),
(3) When working outdoors at air temperatures below 10 °C or above 25 °C,
(4) When working in physically demanding jobs (effective energy expenditure above 1500 kcal for men and 1000 kcal for women),
(5) At workstations where the air temperature exceeds 28 °C.

In summary, under Polish law, employers must provide drinking water to all employees working under normal working conditions without restrictions. However, when working under particularly strenuous conditions (WBGT > 25 °C), the employer must provide other beverages (enriched with minerals and vitamins) in addition to water.

Work is currently underway to standardize and formalize protective measures regarding the Heat Injury and Illness Prevention Standard [163].

Although this work is still ongoing, the most important fact is that the U.S. government recognizes the need to also regulate proper hydration in occupational health and safety regulations.

This document [164] contains the most important general requirements regarding work organization, such as providing breaks for rest in the shade in appropriate quantities and frequencies, depending on the risk of overheating.

The document also addresses the need for training and education for workers and employers on the possible risks of dehydration caused by working in high temperatures. Workers should be trained on topics such as the importance of maintaining hydration (consuming water or electrolytes), knowledge of the right to breaks and water, and requirements for specific procedures (taking into account, for example, work intensity, environmental conditions, age, body composition, and weight). According to the document [164], workers should know how much and what types of beverages they should consume at work. As part of dehydration prevention, workers should self-assess their hydration status using physiological methods, such as the number of visits to the bathroom during a shift (at least once) and urine color. Designated individuals (such as supervisors and heat safety coordinators) should implement and monitor a dehydration prevention plan, for example, by observing workers to ensure they drink water frequently or take breaks. This document also highlights the importance of acclimatizing workers through gradual adaptation to heat conditions. Such acclimatization is an important preventive measure that reduces susceptibility to heat-related illnesses and dehydration.

Therefore, the document will require employers to implement a system for preventing the effects of working in hot conditions—including a plan, monitoring, water, breaks, training, and documentation.

Table 5 summarizes hydration recommendation for employers and workers, especially those working in a thermal microclimate.

It should be remembered, however, that these are general recommendations. Each worker should develop their own individualized hydration plan (depending on the amount of sweat lost and sensation of thirst) to best compensate for fluid losses during work without any gastrointestinal discomfort [60]. In addition, women have more difficulty adapting to a hot environment than men, which requires special attention.

## 9. Summary

Based on the literature review, universal recommendations have been developed for workers to maintain adequate hydration of the body. However, no single recommendation can be applied to all workers, so each individual should create a personalized fluid intake strategy [124,125,126,127].

It should be kept in mind that the pattern of fluid intake is largely habitual and therefore susceptible to modification. Creating an appropriate hydration awareness culture among workers is an important element of risk management strategies for workers exposed to stressful thermal conditions [89].

To effectively prevent dehydration at work, workers should [1,2,68,115,137]:

- Be properly hydrated before starting work;
- Frequently drink small amounts of fluids right from the beginning of work;
- Compensate for fluid deficiencies after finishing work;
- Create an individualized hydration plan.

Moreover, workers should drink small amounts of fluids frequently (no more than 1.5 L/h) and replace lost electrolytes with their diet [45,108,118].

Research to date highlights the role of hydration and its impact on workers. Proper hydration not only satisfies basic physiological needs but also improves mental clarity, reduces fatigue, improves work performance, and supports long-term health. To ensure workers are properly hydrated, employers should consider strategically placing water stations, scheduling adequate hydration breaks, and implementing workplace health education. The synergy between balanced nutrition and proper hydration is a key, yet often overlooked, element of a workplace wellness strategy. By ensuring proper hydration, employers create a more supportive and health-promoting work environment, benefiting both workers and the organization [20].

Despite the clear benefits of ensuring workers are properly hydrated, this topic remains under-researched. There is a lack of research on developing specific recommendations for individual professions based on workers’ hydration status and fluid loss during work, determining the impact of dehydration on the health, performance, and well-being of workers in specific professions; the impact of physical, social, and environmental factors related to fluid intake; and the development of dehydration, both at the micro (days) and macro (weeks, months, years) scales, on worker health outcomes and productivity [19]. This research should be conducted for men and women. Furthermore, future research should also focus on the types of fluids consumed and their impact on health and performance as well as on beverages intended for workers (with an emphasis on appropriate ingredient selection). It is also worth considering the development of sensors for continuous monitoring of fluid and electrolyte loss to develop individualized hydration plans.

### Limitations

A narrative review may be subject to some bias, a lack of objectivity, and subjective interpretation of the results by the authors. However, the authors of this article have attempted to present the problem of dehydration as objectively and comprehensively as possible, and their conclusions are based on a comprehensive review of the scientific literature.

## Figures and Tables

**Table 1:** Percentage of dehydrated workers from various industries before and after work.

Authors	Participants/Workers	Before Work Dehydration (%)	After Work Dehydration (%)	Dehydration Criteria Based on Urine Specific Gravity (USG)
Ueno et al., 2018 [94]	Construction workers, Japan	5%	21%	USG > 1.030
Al-Bouwarthan et al., 2020 [97]	Construction workers, Saudi Arabia	85%	81%	USG ≥ 1.020
Wesseling et al., 2016 [93]	Construction worker (CW), small-scale farmer (SSF), sugarcane cutter (SC), Nikaragua	29% (CW) 20% (SSF) 15% (SC)	No data	USG ≥ 1.030
Mizelle et al., 2022 [98]	Latino farmworkers	47% 0%	92% 8%	USG ≥ 1.020 USG > 1.030
Abasilim et al., 2024 [18]	Farmworker, Mexico, Guatemala	62%	97%	USG > 1.020
Mix et al., 2018 [95]	Agricultural workers, Mexico, Guatemala, Haiti, USA, other	53% 3%	81% 13%	USG ≥ 1.020 USG > 1.030
Biggs et al., 2011 [26]	Forestry workers harvesting trees, South Africa	43% autumn 47% winter	64% autumn 63% winter	USG > 1.020
Orysiak et al., 2022 [24]	Foresters, Poland	70% summer 57% autumn 68% winter	45% summer 48% autumn 63% winter	USG ≥ 1.020
Piil et al., 2018 [99]	Manufacturing workers (indoor aluminum extrusion), agricultural workers, police officers, tourism workers, and construction workers, Denmark, Cyprus, Greece, and Spain	70% (range 44–92% across the five different industries)	69% (range; 14–100% across the five different industries)	USG ≥ 1.020
Polkinghorne et al., 2013 [91]	Underground mines, Australia	59%	58%	USG > 1.020
Kase et al., 2022 [100]	Nurses, Japan	51%	71%	USG ≥ 1.020
Mears and Shirreffs, 2015 [67]	Teacher (T) Security staff (SS) Firefighter (F) Office worker (OW) Catering staff (chefs and kitchen assistants) (CS) Great Britain	39% (T) 60% (SS) 73% (F) 47% (OW) 38% (CS)	23% (T) 53% (SS) 36% (F) 20% (OW) 38% (CS)	USG > 1.020

**Table 2:** Symptoms of dehydration [101,102,103].

	Mild-to-Moderate Dehydration	Severe Dehydration
General appearance/condition	Sleepiness or tiredness Muscle weakness Headache Dizziness Light-headedness	Irritability Feeling unusually tired (lethargic) or confused
Thirst	Feeling thirsty	Extreme thirst
Tears, eyes	Few or no tears when crying Normal or sunken eyes	No tears Deeply sunken eyes
Mucous membranes, skin	Dry, sticky mouth Dry, cool skin	Very dry mouth, skin, and mucous membranes Shriveled and dry skin (lack of elasticity)
Heart rate, respiration	Normal or low blood pressure Normal or deep breathing	Low blood pressure Rapid heartbeat and breathing
Urine output	Less frequent urination	Little or no urination and changing color (dark yellow or amber)

**Table 3:** Selected dehydration effects [8,9,35,104,105].

Percent of Dehydration	Effects
1–2%	Impaired manual labor productivity
	Impaired thermoregulation during exercise
2–3%	Degraded aerobic performance, particularly in warmer environments
	Impaired mental function (visual-motor tracking, short-term memory, attention, and arithmetic efficiency)
	Impaired cognitive/mental/motor function—greater tiredness (fatigue), reduced alertness, and higher levels of perceived effort and concentration in temperate-warm-hot environments
	Vague discomfort
3–8%	Increased effort of exercise; reduced exercise capacity; impaired endurance performance; decreased ability to execute sport-specific skills
	Difficulty concentrating; headache; impatience; apathy
	Impaired reaction time
	Severe impairment in exercise thermoregulation and risk of heat stroke at 6% dehydration
	Risk of collapse at 7% dehydration
8–15%	Dizziness
	Mental confusion
	Spastic muscle, general incapacity, delirium, and wakefulness
15%	Circulatory failure and death

**Table 4:** Examples of different fluid consumption strategies [119,120].

Strategies	Description
Planned drinking	Drinking specific amounts of fluids to minimize fluid losses
	Following an individual fluid replacement plan due to the variability in sweat rate and sweat electrolyte concentration among individuals
	Goal—to prevent dehydration and excessive drinking (±2% of body mass) by drinking an amount of fluid corresponding to the amount of water lost through sweat
Drinking to thirst	Drinking when thirsty
	In most cases, “drinking to thirst” is used as a synonym for “drinking ad libitum” or “planned drinking”
	Goal—to use the innate thirst mechanism to manage fluid intake and prevent the development of exercise-related hyponatremia and excessive dehydration
Ad libitum drinking	Drinking fluids without a specific time or amount—anytime and in any amount
	“Drinking to thirst” may or may not produce the same results as “drinking ad libitum”
	Some experts use “ad libitum” to refer to “planned drinking”

**Table 5:** Recommendations for hydration in the workplace [2,3,8,17,19,22,23,26,27,35,36,54,62,63,64,65,68,69,70,75,76,77,78,79,82,83,84,85,89,90,108,112,115,117,118,124,125,126,134,135,136,137,139,140,149,154,165,166,167].

Scope of Activity	Recommendations
EMPLOYERS SHOULD	
Hydration education	Take care of the proper hydration education of workers Make sure that workers know how to monitor their own hydration status Education should include, e.g., daily fluid needs, types of fluids to optimize hydration, health behaviors, and self-assessment of hydration status.
Adequate hydration in the workplace	Provide workers with constant and easy access to water or other fluids (with a temperature adapted to the environmental conditions) Ensure, that water temperature is 10–15 °C in hot conditions, and warmer in cold environments Provide carbohydrate and/or electrolyte drinks when working in difficult thermal environments and during prolonged physical exertion (>2 h). Create drinking strategies for workers to optimize hydration, minimize body mass loss, promote light/pale urine color, moderate urine frequency (i.e., >5 voids per 24-h), prevent overdrinking, and reduce thirst sensation.
Palatability of drinks	Make sure which drinks are preferred by workers (e.g., bottled water preferred at home and at work over tap water) Ensure that fluids taste pleasant and are odorless
Meals	Encourage workers not to skip meals to replace water and electrolyte losses
Rest breaks	Encourage workers to take rest breaks and drink during breaks Promote (or encourage) fluid intake among workers even if it requires additional breaks Pay attention to women who may avoid breaks due to concerns about appearing weak or when their hourly income is lower
Minimize water loss in the body	Consider changing the organization of work (e.g., working hours in hot and dry environments, working in the evening or night shifts) and/or work clothes and/or increasing frequency of fluid replacement breaks
Acclimatization	Take care of proper acclimatization of workers
Access to toilets	Provide free unlimited access to toilets
Come to work well hydrated	Encourage workers to come to work well hydrated
WORKERS SHOULD	
Hydration education	Take part in hydration educational programs Monitor their own hydration status (urine color, thirst, body mass)
Adequate hydration in the workplace	Drink small amounts of fluids frequently (do not exceed 1.5 L/h or 12 L/day) Create personalized/individual fluid intake strategies depending on, e.g., sunlight exposure, level of physical activity, air temperature, sweating rate, and clothing Drink enough fluids to use the toilet at least once during their shift and maintain pale/light yellow urine color Do not overhydrate—drinking too much water or other fluids may cause medical emergencies
Palatability of drinks	Choose drinks they like to consume (e.g., still or sparkling water) Occasionally choose a different drink with a different temperature (or flavor) to increase voluntary fluid intake Prepare their own drinks (e.g., add sliced citrus or fresh mint) rather than buying ready-made flavored waters Limit or avoid intake of sugary drinks and swap them for no-added-sugar versions
Meals	Eat regularly—replace water and electrolyte losses
Rest breaks	Regularly take rest breaks—time to drink
Minimize water loss in the body	Dress appropriately and/or frequently drink small amounts of fluid
Acclimatization	Take part in acclimatization programs
Come to work well hydrated	Have a bottle of water at work and outside and start replacing fluid losses while driving home Drink fluids throughout the day combined with meals containing sodium

## Data Availability

No new data were created or analyzed in this study. Data sharing is not applicable to this article.
